# METTL3-mediated RanGAP1 promotes colorectal cancer progression through the MAPK pathway by recruiting YTHDF1

**DOI:** 10.1038/s41417-024-00731-5

**Published:** 2024-01-24

**Authors:** Rui Yang, Cheng Yang, Danjie Su, Yang Song, Jie Min, Zhixin Qian, Xiangjing Shen, Junqiang Li, Haichuan Su

**Affiliations:** 1 Department of Oncology, Tangdu Hospital, Air Force Medical University, Xi’an, 710038 Shaanxi China; 2 Department of Obstetrics and Gynecology, Tangdu Hospital, Air Force Medical University, Xi’an, 710038 Shaanxi China

**Keywords:** Colorectal cancer, Cancer

## Abstract

Ran GTPase activating protein 1 (RanGAP1) has been implicated in various diseases, but its role in colorectal cancer (CRC) progression remains unclear. Using tumor tissues and public databases, we found that RanGAP1 was significantly upregulated in CRC tissues and was associated with poor prognosis of patients. N6-methyladenosine (m6A) was found to play an important role in higher expression of RanGAP1. MeRIP-seq, RIP-qPCR, Luciferase reporter assays and other related experiment elucidated the molecular mechanism underlying m6A modification of RanGAP1. Besides, cell function experiments and xenograft tumor models corroborated the function of RanGAP1 in CRC progression. By RNA-seq and related analysis, RanGAP1 was verified to influent CRC progression via the Mitogen-Activated Protein Kinase (MAPK) signaling pathway. Therefore, N6-methyladenosine modification of RanGAP1 by METTL3/YTHDF1 plays a role in CRC progression through the MAPK pathway and could be a potential biomarker and therapeutic target for CRC.

**Figure:**
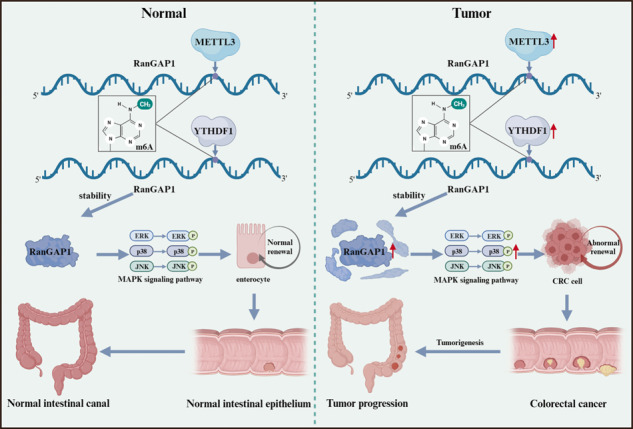
Schematic diagram showed that N6-methyladenosine modification of RanGAP1 promotes CRC progression via the MAPK signaling pathway.

Schematic diagram showed that N6-methyladenosine modification of RanGAP1 promotes CRC progression via the MAPK signaling pathway.

## Introduction

Over the past five years, colorectal cancer is still the third most common cancer worldwide, causing an extremely high death rate [1]. Due to the recent vigorous development of colonoscopy and other procedures, the prevention, diagnosis, and treatment of colorectal cancer have significantly progressed. However, because of its rapid proliferation and frequent metastasis, the prognosis of patients is still less than satisfactory [2]. Hence, it’s extremely important to further explore the molecular mechanism of CRC to improve treatment strategies.

RanGAP1 is the GTPase activating protein of the small GTPase Ran, which is primarily localized in the cytoplasm [3]. The RanGAP1-SUMO1 protein is localized in the nuclear membrane and functions as a component of the cytoplasmic filaments within the nuclear pore complex (NPC), regulating nuclear transport [4, 5]. Additionally, the RanGAP1/RanBP2 complex plays a crucial role in ensuring stable kinetochore-microtubule association [6]. In osteosarcoma, the loss of RanGAP1 leads to chromosomal instability and rapid tumorigenesis [7]. Nevertheless, the expression level and the role of RanGAP1 in CRC remain ambiguous.

N6-methyladenosine is currently considered as one of the most common forms of transcript modification in eukaryotes. The m6A methyltransferase METTL3 (a writer) and the specific RNA binding protein YTHDF1 (a reader) can directly or indirectly identify and bind specific m6A motifs to regulate mRNA stability or protein translation [8]. Previous studies have shown that METTL3 and YTHDF1 are involved in CRC progression. However, their biological significance and potential regulatory mechanism remain unclear.

The protein cellular retinoic acid-binding protein 2 (CRABP2) acts as a transporter, shuttling between the cytosol and nucleus, to deliver the vitamin A metabolite retinoic acid (RA) to nuclear receptors RAR and RXR [9]. RA is a potent regulator of cell growth and differentiation.

In our research, we demonstrated that RanGAP1 exhibited significant upregulation in CRC and served as a potential downstream effector of METTL3 and YTHDF1, undergoing m6A methylation modification to exert regulatory effects on the expression levels of RanGAP1. Moreover, RanGAP1 exerted its impact on disease progression through the regulation of CRABP2 and the MAPK signaling pathway. These findings suggested that RanGAP1 held promise as a potential biomarker and therapeutic target for CRC.

## Materials and methods

### Colorectal cancer patient samples

Cancer and para-cancer tissue samples were collected from CRC patients at Tangdu Hospital of Air Force Medical University who met the diagnostic criteria for CRC according to the 2020 revised Joint American Council on Cancer (AJCC) manual and provided informed consent. The study was approved by the Ethics Committee of Tangdu Hospital of Air Force Medical University, and all relevant tissues were stored in liquid nitrogen. The data from TCGA and GEO databases pertaining to CRC and normal samples have been presented in Table S1.

### Cell culture and lentivirus infection

All cells used in this study were purchased from Procell Life Science & Technology Company (Wuhan, China) with STR authentication. The cells were identified to be free from mycoplasma contamination. HCT116 cells were cultured in McCoy’s 5 A medium supplemented with 10% FBS, while DLD1 cells were cultured in RPMI medium (HyClone Laboratories Inc, Logan, UT, USA). The packaging plasmids (pMD2G and psPAX2) were purchased from Vigene Biosciences (Jinan, China). For packaging lentivirus: 293 T cells were cultured on 100-mm plates at a concentration of 7 × 10^6^ cells per dish. Twenty-four hours later, the serum-free medium with a mixture of lentivirus plasmid (15 µg), pMD2G (4.5 µg), psPAX2 (9 µg), and Lipofectamine 3000 (46 µg; Invitrogen, Carlsbad, CA, USA) was used to replace the original medium. The complete medium was replaced after 10 h, and the supernatant was collected 24 h later. The lentivirus was obtained by filtering the supernatant through a 0.45-µm filter membrane. For infecting cell, cells were placed in a 100-mm Petri dish at a concentration of 2 × 10^6^ each dish. After 24 h, lentivirus and Polybrene were added and the medium was replaced with uncomplete medium. After 12 h, the fresh complete medium was replaced and the cells were cultured until the density reached 90%. Then the infected cells were screened using puromycin or G418. The shRNA and exogenous RNA sequences about this research are shown in Table S2.

### Proliferation assays

The CCK-8 reagent was obtained from KeyGEN (Jiangsu, China) and used as directed by the manufacturer. The target cells were dissolved in 100 μL medium at a density of 10^3^/well and cultured in 96-well microplates. Analysis of absorbance was conducted at a 450-nm wavelength, and absorbance was continuously monitored at 0, 24, 48, 72, and 96 h to analyze proliferation. The colony formation assay procedures were conducted in accordance with previously established protocols [10].

### Invasion and migration assay

The invasion and migration capability of CRC cells were evaluated using the cell scratch wound-healing assay and transwell assay. In the cell scratch wound-healing assay, cells were cultured in 6-well plates until reaching a density of 100%, followed by creating uniform scratches at the center of each well using micropipette tips. Following removal of deciduous cells with Phosphate-Buffered Saline (PBS) (Servicebio), the remaining anchorage-dependent cells were cultured in incomplete medium and imaged at 0, 12, and 24 h. Migration area (%) was calculated as (1 – A_n_/A_0_), where A_n_ represents the scratch area at different time points. The transwell assay procedures were conducted in accordance with previously established protocols [10].

### RNA extraction and quantitative real-time PCR

RNA was extracted from tissues and cells using Trizol reagent (Ambion Inc, Austin, TX, USA) following the manufacturer’s protocol. Subsequently, cDNA was synthesized with HiScript II (Vazyme Biotech, Nanjing, China) and RanGAP1 expression levels were quantified by qPCR using a Mx3005P instrument (Agilent Technologies, California, USA) and SYBR Green qPCR Master Mix (Servicebio). GAPDH served as the internal control for mRNA analysis. The primer sequences pertaining to the research are presented in Table S3.

### Western blot analysis

Cells and tissues were first lysed using lysis buffer (Applygen, Beijing, China) with a phosphatase and protease inhibitor cocktail (Roche, Branchburg, NJ, USA). Subsequently, sodium dodecyl sulfate-polyacrylamide gel electrophoresis was used to fractionate the different proteins. Polyvinylidene difluoride (PVDF) membranes were blocked with 5% nonfat milk for 3 h and subsequently incubated with antibodies at a temperature of 4 °C for a duration of 10 h. Subsequently, the PVDF membranes were incubated with the appropriate IgG-HRP conjugate and the resulting protein bands were analyzed using a Bio-Rad ChemiDocTM XRS+ imaging system (Bio-Rad Laboratories). All antibodies used for this research are listed in Table S4.

### RNA immunoprecipitation assay

RNA immunoprecipitation was performed using an EZ-Magna RNA-Binding Protein Immunoprecipitation Kit (MilliporeSigma, Burlington, MA, USA) following the manufacturer’s instructions. Initially, target cells were lysed with RIPA buffer containing protease and RNase inhibitors. Subsequently, the lysates were incubated with magnetic beads conjugated with anti-METTL3 (Proteintech) and anti-YTHDF1 (Proteintech) antibodies. After the washing steps, proteinase K was used to digest the complex and obtain immunoprecipitated RNA, which were then purified and analyzed by qRT-PCR.

### Methylated RNA immunoprecipitation sequencing (MeRIP-seq)

Total RNA samples were obtained as described in the preceding sections. The Magna Methylated RNA Immuno-Precipitation (MeRIP) m6A Kit was purchased from MilliporeSigma. For immunoprecipitation, the RNA fragments (100 nucleotides) were incubated with m^6^A antibodies, after which m^6^A methylation enrichment of mRNA was analyzed using high-throughput sequencing.

### Luciferase reporter assay

RanGAP1 fragments with mutant (Mut) or wild-type (WT) binding sites were subcloned into the psiCHECK2 dual-luciferase reporter vector (Hanbio, China) resulting in the following constructs: RanGAP1-WT, RanGAP1-Mut. And the plasmids were co-transfected into HCT116 cells with shMETTL3-1/2 mimics or NC mimics. After 48 h, the Dual-Luciferase Reporter Assay System (E1910, Promega, USA) was used to measure relative luciferase activity. Relevant sequences are listed in Table S5.

### Tissue microarray and immunohistochemistry (IHC)

The tissue microarray kits for CRC patients were acquired from Outdo Biotech Company (HColA180Su10, Shanghai, China) (https://www.superchip.com.cn/). After antigen repair and blocking, the tissue microarray and sections were incubated with RanGAP1 or CRABP2 antibodies overnight at 4 °C. Subsequently, the tissue microarray and sections were subjected to a 30-minute incubation with secondary antibodies, followed by staining with diaminobenzidine for 3 min and counterstaining with hematoxylin. The scoring of IHC was as follows: positive cell ratio = number of positive cells/total number of cells. Positive cell density = number of positive cells/tissue area to be measured. Average optical density value = cumulative optical density IOD value/positive pixel area. H-SCORE = ∑ (pi × i) = (percentage of weak intensity × 1) + (percentage of moderate intensity × 2) + (percentage of strong intensity × 3).

### Flow cytometry analysis

The cells were initially cultured in 6-well plates (10^6^ cells per well) in triplicate for 24 h and synchronized at the G0/G1 phase by replacing uncomplete medium for 8 h. Afterwards, replacing complete medium (with 10% FBS) to continue culturing for 24 h. Subsequently, the cells were digested, washed three times by PBS, and fixed in precooled 70% ethanol overnight in 4 °C. Finally, the cells were stained by PI/RNase staining buffer (BD Biosciences) and subjected to flow cytometry using a BD FACSCalibur system (BD Biosciences) to analyze the cell cycle.

### Label-free quantitative LC/MS proteomics and co-immunoprecipitation mass spectrum

RanGAP1-silenced CRC cells and control cells were cultured in triplicate in 100 mm culture dishes. Once the cell density reached 90%, they were enzymatically dissociated using trypsin and subsequently washed three times with PBS. Subsequently, Mhelix Biotech Company (Shanghai, China) conducted label-free quantitative liquid chromatography-mass spectrometry (LC/MS) proteomics analysis on the cells. The Co-Immunoprecipitation Mass Spectrum assay procedures were conducted in accordance with previously established protocols [10].

### Xenografts in mice

The nude mice experiment in this study was approved by the Ethics Committee of Experimental Animal Center of the Air Force Medical University. The mice were categorized using a random number table methodology. Cells stably transfected with shNC and shRanGAP1 were injected subcutaneously into the back of female athymic BALB/C nude mice aged 4–6 weeks at a concentration of 5 × 10^6^ cells/mouse. Every 3 days, tumor volume was measured (volume = longest diameter × shortest diameter [2] × 0.5). After 4 weeks, the mice were euthanized, tumors weighed and sections made for immunohistochemical staining.

### Statistical analysis

All the experiments were repeated at least three times. Data analysis was conducted in SPSS 19.0 (SPSS Inc, Chicago, IL, USA). The data were presented as mean ± SD. Paired/unpaired Student’s *t*-tests were used to analyze differences between two samples in paired/unpaired results. The χ [2] test was employed to examine the relationship between mRNA/protein levels and clinicopathological parameters. The Kaplan–Meier curve was used to examine the correlation between mRNA/protein levels and overall survival and disease-free survival. Statistical significance was defined as *p < 0.05, **p < 0.01, ***p < 0.001 and ****p < 0.0001.

## Results

### RanGAP1 is highly expressed in CRC

**Fig. 1:**
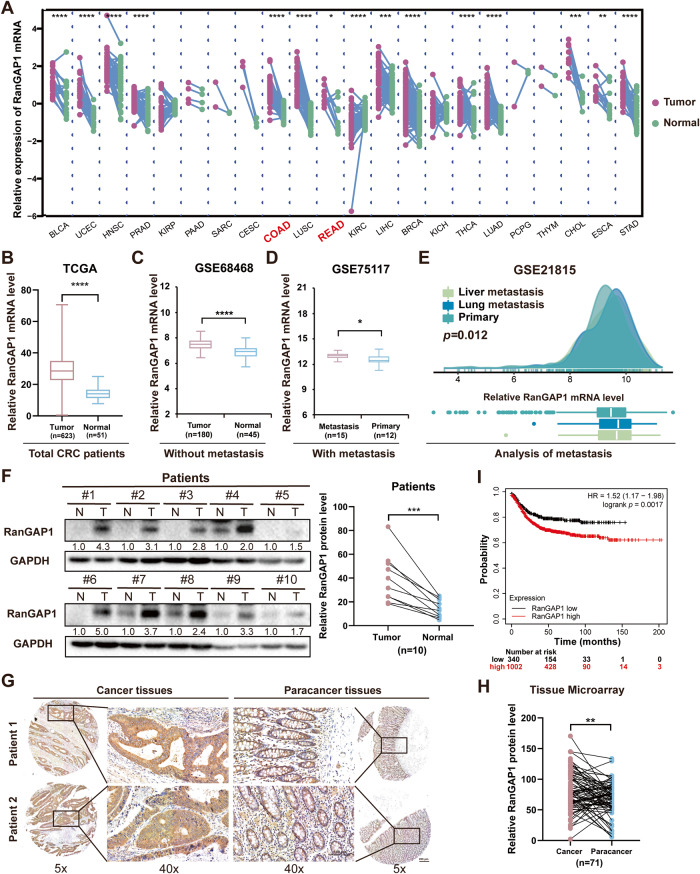
RanGAP1 is highly expressed in CRC and considered as a diagnostic indicator. **A** RanGAP1 mRNA expression levels were higher in multiple cancers than in normal tissues based on TCGA data. **B**, **C** The mRNA expression levels of RanGAP1 based on TCGA and GSE68468 data. **D**, **E** RanGAP1 mRNA expression levels of patients with primary and metastatic colon cancer based on GEO datasets. **F–H** The protein expression levels of RanGAP1 were compared between CRC tissues and matched para-cancer tissues (*n* = 10), and were subsequently analyzed in Tissue Microarray samples of CRC and normal tissue. **I** The survival analysis between RanGAP1 expression and Recurrence free survival (RFS) of CRC based on KMplot data.

To assess the expression level of RanGAP1 in cancer, we utilized The Cancer Genome Atlas (TCGA) database and observed elevated levels of RanGAP1 in several cancers, including Colon Adenocarcinoma (COAD) and Rectum Adenocarcinoma (READ) (Fig. 1A, B, Fig. S1A). Furthermore, we conducted an analysis of various stages of CRC and observed that non-metastatic CRC patients exhibited a significantly elevated mRNA level of RanGAP1 in tumor tissues based on the GSE68468 (Fig. 1C) and GSE49355 (Fig. S1B). Our findings were further corroborated by metastatic CRC patients from GSE35834 dataset (Fig. S1C). Subsequent analysis of metastatic CRC revealed that the levels of RanGAP1 mRNA in primary tumors were higher in patients with metastasis compared to those without (Fig. 1D, Fig. S1D, E). Furthermore, examinations of the liver and lungs, which are the most common sites for CRC metastasis, demonstrated that RanGAP1 mRNA levels were elevated in CRC patients with distant metastases to these organs compared to those without metastasis (Fig. 1E). Moreover, this conclusion was validated at the protein level. Tissue samples from 10 CRC patients demonstrated significantly elevated levels of RanGAP1 protein expression in tumor tissues compared to para-cancerous tissues (Fig. 1F). Additionally, results from the CPTAC database also supported this finding (Fig. S1F). Immunohistochemistry of CRC patient tissue microarray samples also verified that RanGAP1 expression levels were considerably improved in CRC samples compared with those in control samples (Fig. 1G, H). The KMplot (www.kmplot.com) database revealed a significant association between elevated levels of RanGAP1 in CRC and poorer Recurrence Free Survival (RFS) (Fig. 1I). In conclusion, RanGAP1 might serve as a potential biomarker for CRC diagnosis.

### RanGAP1 promotes CRC tumorigenesis in vitro and in vivo

**Fig. 2:**
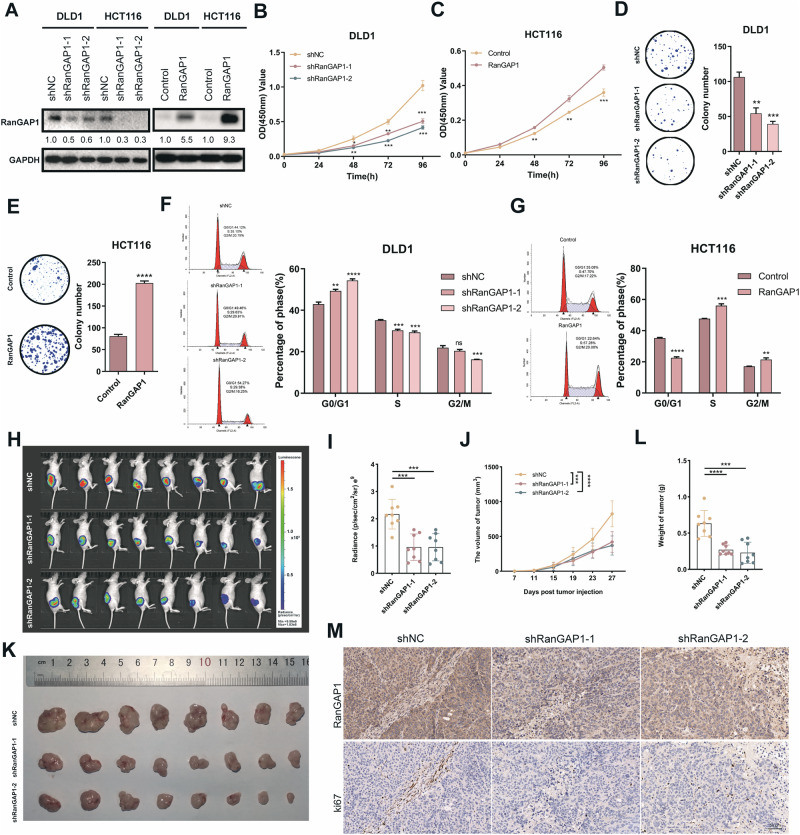
RanGAP1 promotes proliferation of colorectal cancer. **A** The intervention effect of RanGAP1 knockdown and RanGAP1 overexpressed in CRC cells. **B–E** CCK8 assays and colony formation assays showed the proliferation ability of silencing and overexpressing RanGAP1 on CRC cells. **F**, **G** Flow cytometry assays showed the effects of silencing and overexpressing RanGAP1 on the cycle of CRC cells. **H**, **I** Injection of modified RanGAP1 knockdown DLD1 cells in nude mice. **J** Volume of tumors were recorded every four days. **K** Xenografts from nude mice were measured. **L** Weight of tumors in each group. **M** The proteins levels of RanGAP1 and ki67 were quantified.

To explore the potential role of RanGAP1 in CRC, RanGAP1 overexpression and knockdown CRC cell lines were established by lentiviral infection (Fig. 2A). Compared to the control groups, knockdown of RanGAP1 significantly reduced cell proliferation abilities in CRC cell lines as demonstrated by CCK8 and colony formation assays, while overexpression of RanGAP1 remarkably increased cell viability (Fig. 2B–E, Fig. S2A–D). Additionally, the analysis of flow cytometry revealed a significant increase in the percentage of cells in G0/G1 phase and a noticeable decrease in those in S phase upon knockdown of RanGAP1 in CRC cells (Fig. 2F, Fig. S2E). In addition, RanGAP1 overexpression notably elevated the percentages of the S phase in HCT116 (Fig. 2G) and DLD1 cells (Fig. S2F), confirming that RanGAP1 significantly promotes CRC cell growth. To further prove our conclusion in vivo, we used nude mice to perform xenograft assays. Subcutaneous tumors were compared using animal imaging on day 27, and consistent with the results in vitro, the fluorescence intensity of the shRanGAP1 group was lower (Fig. 2H, I). Additionally, the tumor volume of the shRanGAP1 group increased more slowly (Fig. 2J). Besides, the weight of shRanGAP1 group subcutaneous tumors was statistically lower than control (Fig. 2K, L, Fig. S2G), which also verified our conclusion. Moreover, IHC indicated that the expression of Ki67 in RanGAP1 knockdown tumor tissues was observably lower (Fig. 2M).

**Fig. 3:**
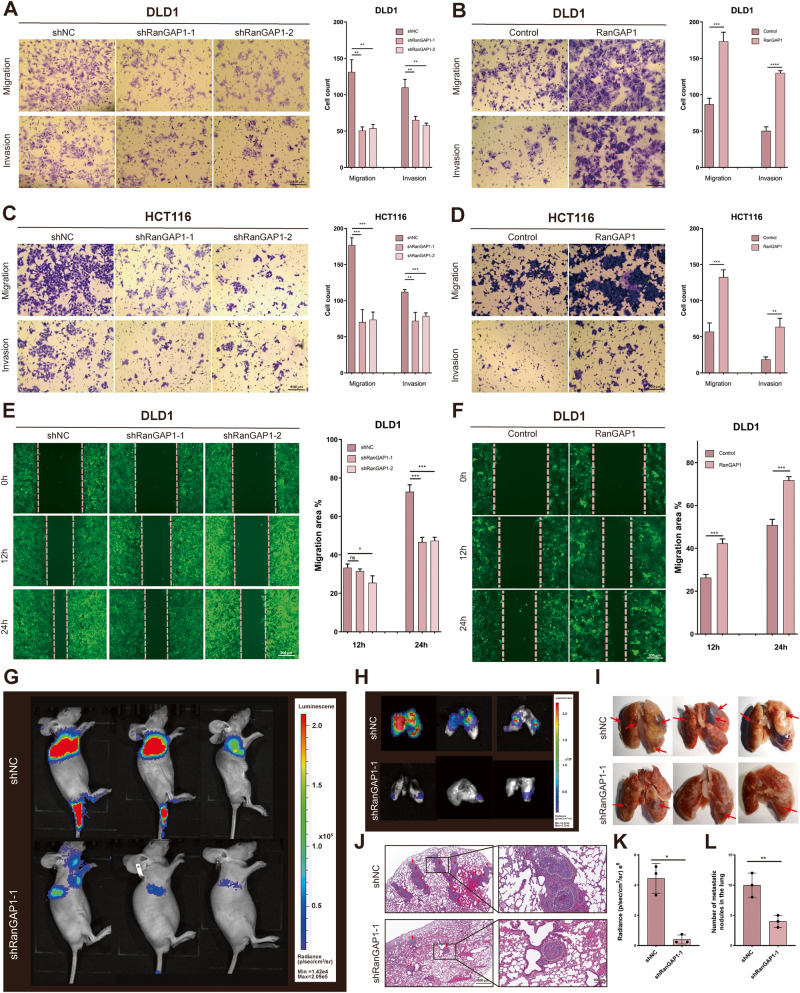
RanGAP1 promotes metastasis of CRC. **A–F** The migration and invasion abilities were analyzed in RanGAP1 knockdown and overexpressed CRC cells. **G** Representative mice injected with RanGAP1 knockdown DLD1 cells and control cells were measured for CRC lung metastases. **H**, **I** Lung from nude mice were obtained and analyzed. **J–L** The HE-staining detected the expression of the metastatic nodules of lung in the different groups.

The results showed that RanGAP1 knockdown remarkably reduced migration and invasion capacity of CRC cells by transwell and scratch wound assays (Fig. 3A, C, E). Contrastingly, overexpressed RanGAP1 remarkably increased migration and invasion capacity of CRC cells (Fig. 3B, D, F). To sum up, these findings indicated that RanGAP1 accelerates the migration and invasion of CRC in vitro. In vivo, we used the tail vein metastasis experiment to confirm this hypothesis and showed that RanGAP1 knockdown significantly decreased the incidence of lung metastasis and reduced the number of metastatic lung nodules (Fig. 3G–L), consistent with the results in vitro.

### METTL3-mediated m6A modification of RanGAP1 maintains its stability in CRC

**Fig. 4:**
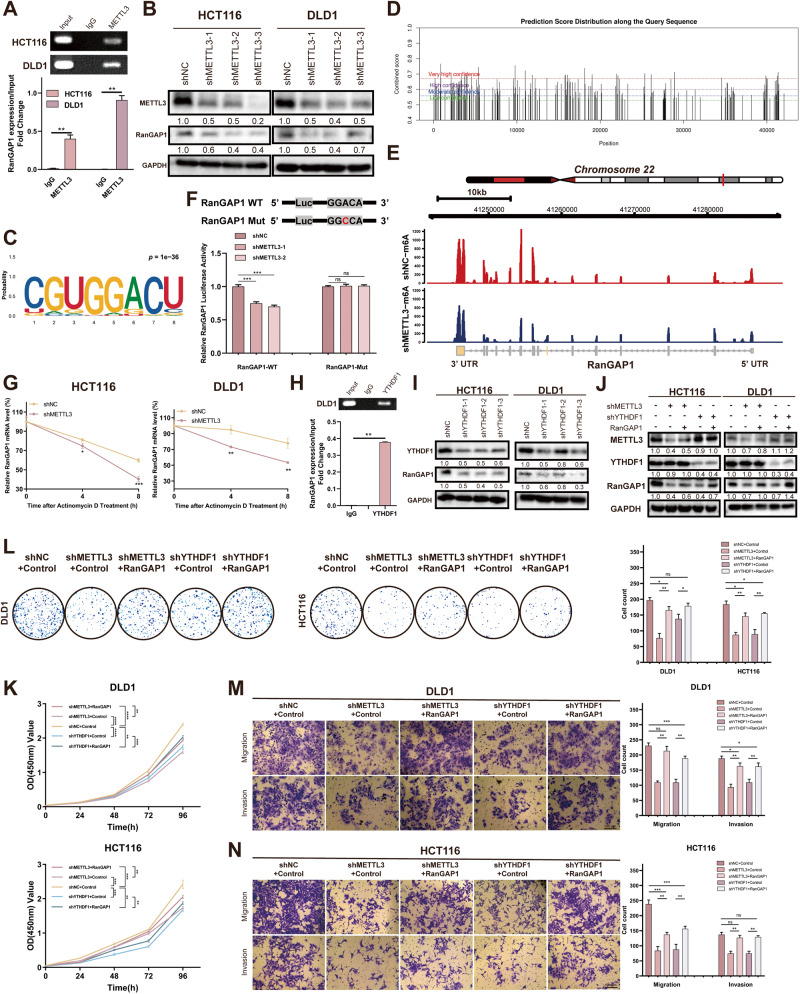
METTL3 regulates RanGAP1 by recruiting YTHDF1 to promote the progression of CRC. **A** RIP assays showed the binding between the METTL3 protein and RanGAP1 mRNA. **B** The protein levels of RanGAP1 in METTL3 knockdown CRC cells. **C** m6A motifs were identified by MeRIP-seq in METTL3 silenced HCT116 and control group. **D** SRAMP website showed the potential site of m6A modification of RanGAP1 mRNA. **E** METTL3 knockdown reduced m6A modification of RanGAP1 mRNA in HCT116 cells. **F** Luciferase reporter assays showed the m6A modification affect the expression of RanGAP1. **G** RNA stability of RanGAP1 mRNA in downregulating METTL3 CRC cells and control cells after giving actinomycin D (5 μg/mL). **H** RIP assays showed the binding between the YTHDF1 protein and RanGAP1 mRNA in DLD1 cells. **I** The protein levels of RanGAP1 in YTHDF1 silenced CRC cells. **J** The protein expression levels in CRC cells transfected stably with the shNC+Control, shMETTL3+Control, shMETTL3+RanGAP1, shYTHDF1+Control and shYTHDF1 + RanGAP1. **K–N** The downtrend of proliferation, migration and invasion by shMETTL3 or shYTHDF1 of HCT116 and DLD1 were sectionally recurred because of RanGAP1 overexpressing.

In order to investigate the potential mechanisms regulating RanGAP1 expression, we utilized the starBase v2.0 (https://rnasysu.com/encori/) database for predicting the RNA-binding proteins (PBPs) associated with RanGAP1 mRNA (Table S6). we performed enrichment analysis using the STRING website (https://cn.string-db.org/), which revealed a significant association between RanGAP1 mRNA and m6A modification (Fig. S3A). The dynamic and reversible regulation of RNA m6A modifications is mediated by two key catalytic proteins, named methyltransferases and demethylases [11]. Several classic methyltransferases, including METTL3, METTL5, METTL14 and METTL16 were examined in CRC through GEPIA website (http://gepia.cancer-pku.cn/) [12]. The expression of METTL3 was not only significantly upregulated in colorectal cancer and associated with a poor prognosis in patients but also showed the positive correlation with RanGAP1 across TCGA, KMplot, GSE39582 and GSE161158 datasets (Fig. S3B–E). Next, we validated that RanGAP1 mRNA can be combined directly with METTL3 in CRC cell lines (HCT116 and DLD1) via RIP-qPCR (Fig. 4A). Previous studies [13–17] and our current investigation have demonstrated the involvement of METTL3 in CRC progression (Fig. S3F–I).

In order to further investigate the mechanism underlying METTL3’s regulation of RanGAP1, we confirmed the positive regulation of RanGAP1 by METTL3 at the protein level in HCT116 and DLD1 (Fig. 4B). Subsequently, to investigate whether METTL3 mediates m6a modification on RanGAP1 mRNA, we conducted m6A-modified RNA immunoprecipitation sequencing (MeRIP-seq) on HCT116 cells with silenced METTL3 and control cells. The results showed the RanGAP1 m6A modification level for RanGAP1 decreased 2.88-fold upon METTL3 knockdown (*p* < 0.05). M6A methylation often satisfies the RRACH (R = G or A, H = A, C or U) consensus sequence in transcripts. The results of MeRIP-seq showed that the GGACU motif was highly enriched in the immunopurified mRNA of our sample (p = 1e-36) (Fig. 4C), and most of the m6A-related peak reduction caused by METTL3 knockdown was concentrated in the 3′ and 5′ UTR regions of mRNA (Fig. S3J). We utilized the SRAMP website (http://www.cuilab.cn/sramp/) to predict the mRNA sequence of RanGAP1 and identified numerous potential high-confidence m6A modification sites within it (Fig.4D). Combined MeRIP-seq with predicted results, we determined that the m6A modification site of RanGAP1 might be located in the GGACA region of the 3′ UTR region (Fig. 4E, Fig. S3K). Next, we used Luciferase reporters to verify the accuracy of the predicted site and constructed a RanGAP1 variant by replacing the adenosine in m6A consensus sequences (GGAC) with cytosine. We found that the mRNA levels of wild-type RanGAP1 remarkably decreased after METTL3 knockdown, but its variation was not reduced (Fig. 4F). Additionally, the stability of RanGAP1 mRNA was found to be reduced following METTL3 knockdown, as demonstrated by treating CRC cells with a transcription inhibitor (actinomycin D) for a specified period (Fig. 4G, Fig. S3L).

Taken together, METTL3 regulates the mRNA level of RanGAP1 through m6A modification, thus affecting its protein expression.

### METTL3 recruits YTHDF1 to regulate RanGAP1 mRNA stability

The N6-methyladenosine modification typically requires a “reader” to facilitate METTL3-mediated upregulation of RanGAP1 expression. Previously, researchers have reported the presence of m6A “readers”, including IGF2BP1/2/3, YTHDF1/2/3, and YTHDC1/2 [11]. The correlation between these “readers” and RanGAP1 was assessed using the GEPIA website with results indicating that YTHDF1 exhibits the strongest correlation with RanGAP1 (p = 3.7e-15, r = 0.42) (Fig. S4A, B). Subsequent RIP-seq analysis in GSE136664 revealed the interaction between YTHDF1 and RanGAP1 mRNA in HCT116 cells, which was further validated through RIP-PCR experiments in DLD1 cells (Fig. 4H). These findings strongly suggest the potential involvement of YTHDF1 in epigenetic modifications of RanGAP1. Additionally, the western blotting demonstrated that knockdown of YTHDF1 led to a significant reduction in RanGAP1 protein levels (Fig. 4I). Moreover, the RNA decay rate assay demonstrated that RanGAP1 mRNA was more likely to degrade upon YTHDF1 knockdown in DLD1 and HCT116 cells (Fig. S4C, D). Above all, these findings confirmed that YTHDF1 is the “reader” in RanGAP1 mRNA methylation, and it enhanced mRNA stability. Previous studies [18–20] and our current investigation showed that YTHDF1 promotes CRC progression (Fig. S4E-H).

We overexpressed RanGAP1 in METTL3 and YTHDF1 knockdown CRC cells (DLD1 and HCT116) (Fig. 4J), demonstrating that the overexpression of RanGAP1 can partially rescue the tumorigenesis and metastasis functions affected by shMETTL3/YTHDF1 (Fig. 4K-N). In summary, our findings indicated that METTL3/YTHDF1 facilitates CRC progression via RanGAP1.

### RanGAP1 promotes CRC tumorigenesis via the MAPK signaling pathway

**Fig. 5:**
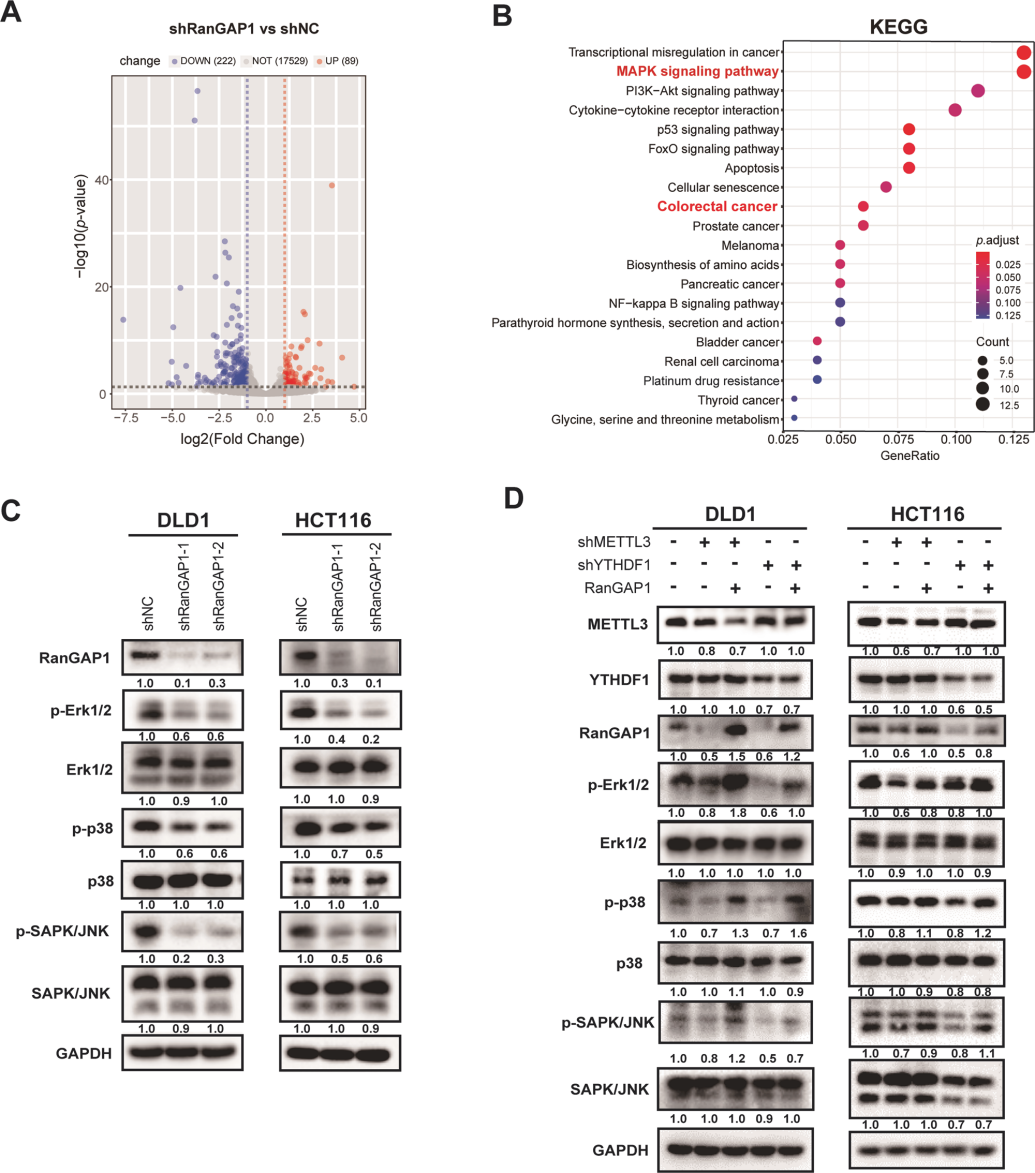
RanGAP1 facilitates CRC tumorigenesis via the MAPK signaling pathway. **A** The volcano plot of differential mRNA expression in RanGAP1 silenced DLD1 and control group ( | log2(fold change) |>1 and *p* value < 0.05). **B** KEGG enrichment analysis of different genes in RanGAP1 knockdown cells and control cells. **C**, **D** Expression levels of proteins associated with the MAPK signaling pathway were evaluated in CRC cells transfected stably with the shRanGAP1, shNC+Control, shMETTL3+Control, shMETTL3+RanGAP1, shYTHDF1+Control and shYTHDF1 + RanGAP1.

To further investigate the potential downstream molecular mechanisms of RanGAP1 in CRC, we conducted whole transcriptome sequencing (RNA-seq) analysis on RanGAP1 knockdown DLD1 cells compared to the control (*n* = 3 in each group). We identified a total of 311 differentially expressed genes (DEGs) between the two groups, with 89 upregulated genes and 222 downregulated genes in the RanGAP1 knockdown cells (*p* < 0.05, |log2FoldChange | > 1) (Fig. 5A). The top 20 Kyoto Encyclopedia of Genes and Genomes (KEGG) pathways of DEGs were investigated, revealing that the MAPK signaling pathway exhibited the highest enrichment level among them (Fig. 5B). The three major subfamilies of MAPK include extracellular-signal-regulated kinases (ERK), c-jun N-terminal kinase or stress-activated protein kinases (JNK or SAPK), and MAPK14 (P38) [21]. To assess the regulatory role of RanGAP1 in the MAPK signaling pathway, we employed a western blot assay to examine the expression levels of Erk1/2, p38, SAPK/JNK and their phosphorylated forms. The results indicated that knockdown of RanGAP1 in CRC cells resulted in a decrease in phosphorylation levels, while the overall abundance of total Erk1/2, p38, SAPK/JNK remained unchanged (Fig. 5C). Additionally, we performed overexpression of RanGAP1 in METTL3-silenced or YTHDF1-silenced CRC cells and observed that the upregulation of RanGAP1 restored phosphorylation levels within the MAPK signaling pathway (Fig. 5D).

### RanGAP1 promotes CRC tumorigenesis via CRABP2

**Fig. 6:**
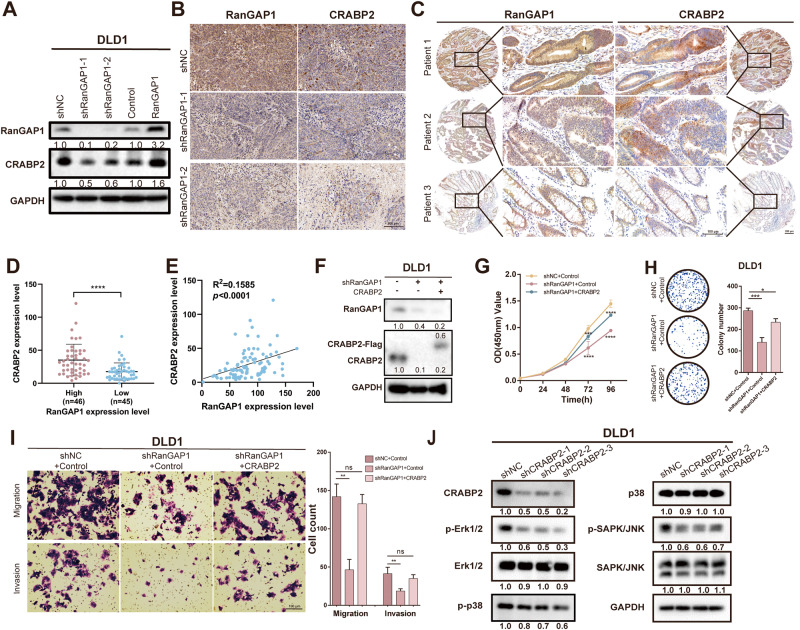
CRABP2, acting as downstream of RanGAP1, is affected by RanGAP1 at protein levels. **A** Western blotting shows change of CRABP2 protein expression levels in RanGAP1 knockdown and RanGAP1 overexpressed CRC cells. **B** RanGAP1 and CRABP2 proteins in xenografts from nude mice are analyzed by IHC (Fig. 2K). **C–E** IHC shows the relationship of RanGAP1 and CRABP2 protein expression in human colon cancer tissue microarray. **F** RanGAP1 and CRABP2 expression levels in CRC cells stably transfected with the shNC+Control, shRanGAP1+Control and shRanGAP1 + CRABP2 by western blotting. **G–I** The downtrend of proliferation, migration and invasion by shRanGAP1 of DLD1 were sectionally recurred because of CRABP2 overexpressing. **J** The protein expression levels associated with the MAPK signaling pathway were assessed in DLD1 cells stably transfected with shCRABP2.

To investigate the underlying mechanism by which RanGAP1 promotes CRC progression through the MAPK signaling pathway, co-immunoprecipitation followed by protein mass spectrometry (MS) was conducted to identify the proteins that interact with RanGAP1 in CRC cells. Label-free LC/MS proteomics and co-IP protein MS identified 12 proteins as candidate RanGAP1-binding proteins (Fig. S5A). Among these 12 proteins, three ribosomal proteins related to protein translation were excluded. We confirmed that the levels of CRABP2 protein significantly decreased in RanGAP1-silenced cells compared to control cells, while overexpression of RanGAP1 resulted in opposite outcomes (Fig. 6A, Fig. S5B). The CRABP2 protein is a cytosol-to-nuclear shuttling protein, which facilitates retinoic acid (RA) binding to its cognate receptor complex and transfer to the nucleus. As a protein associated with the nuclear pore complex, RanGAP1 also regulates the nuclear transport of substances, and it may be functionally related to CRABP2. CRABP2 has been documented to facilitate metastasis in lung cancer by activating the ERK signaling pathway [22]. For further proof, we conducted tissue-level experiments. Immunohistochemistry (IHC) of the excised tumor sections (Fig. 2K), which verified the correlation between proliferation and RanGAP1, indicated that the CRABP2 expression levels in RanGAP1 knockdown tumor tissues were observably lower (Fig. 6B). In addition, the same conclusion was reached in the tissue microarray, and CRABP2 expression was also enhanced in CRC tumor tissues of patients with high RanGAP1 expression (Fig. 6C, D). And a positive association was observed between protein expression levels of RanGAP1 and CRABP2 (Fig. 6E). The previous studies conducted by our team have demonstrated the facilitative role of CRABP2 in the progression of CRC. To confirm whether CRABP2 overexpression could rescue the tumorigenesis and metastasis functions affected by shRanGAP1, we constructed related stable cell lines (Fig. 6F). CCK-8, colony formation, and transwell assays were used to demonstrate that the dampened proliferation, migration, and invasion induced by RanGAP1 knockdown of CRC cells were partially recovered by CRABP2 overexpression (Fig. 6G–I). The Western blot analysis also revealed a decrease in the phosphorylation of the MAPK signaling pathway in DLD1 cells with CRABP2 knockdown (Fig. 6J). To further validate the interaction between RanGAP1 and CRABP2, total proteins were immunoprecipitated using an anti-RanGAP1 antibody. However, co-immunoprecipitation of RanGAP1 and CRABP2 was not observed in DLD1 cells (Fig. S5C). Subsequently, we hypothesized and subsequently confirmed through q-PCR that RanGAP1 is capable of modulating the mRNA expression level of CRABP2 (Fig. S5D). In conclusion, RanGAP1 may exert an influence on the progression of colorectal cancer through its regulation of the MAPK signaling pathway via CRABP2.

## Discussion

Colorectal cancer accounts for 8% of all cancers, and the proportion of young patients has increased annually [23]. Therefore, research on the mechanism of colon cancer continues, and new theories need to be explored in order to find suitable treatments. RanGAP1 encodes a protein that associates with the nuclear pore complex and participates in regulating nuclear transport [24]. The encoded protein interacts with Ras-related nuclear protein 1 (RAN) and regulates guanosine triphosphate (GTP)-binding and exchange [25]. The scaffold protein β-arrestin2 facilitates the translocation of Mdm2 from the nucleus to the cytoplasm in lung and breast tumor cell lines by binding to the RanBP2/RanGAP1-SUMO complex, thereby augmenting p53 signaling [26]. The role of RanGAP1 in hepatocellular carcinoma is demonstrated by its ability to promote cell migration and invasion through the up-regulation of KMD2A expression [27] or LASP1 SUMOylation [28]. We found that RanGAP1 expression was elevated in CRC and promoted its progression.

To investigate the regulatory mechanisms of RanGAP1 expression, Starbase v2.0 and GO enrichment analysis were primarily associated with m^6^A modification. As a “writer” of m6A, METTL3 has been demonstrated to promote CRC progression by exerting influence on amplification [29] and metastasis [16]. Similarly, as a “reader” of m6A, YTHDF1 also plays a crucial role in CRC through an m6A-dependent mechanis [18, 20, 30]. Our study has confirmed that m6A methylation of RanGAP1 by METTL3 is recognized by YTHDF1 and affects its expression. Besides, upregulated RanGAP1 in the METTL3/YTHDF1 knockdown cells could affect phenotype. To further investigate the potential mechanism by which RanGAP1 promotes CRC progression. Through RNA-seq and Western blot analysis, we have identified that RanGAP1 facilitates the progression of colorectal cancer by enhancing the phosphorylation levels within the MAPK signaling pathway. Previous studies have demonstrated that RanGAP1 also exerts its regulatory effects on colorectal cancer progression via the WNT signaling pathway [31]. The MAPK pathway is an intracellular signaling cascade that plays a critical role in regulating cell proliferation, differentiation, apoptosis, inflammation and immunity [32–37]. Our study has demonstrated that the knockdown of RanGAP1 significantly attenuated the phosphorylation levels of Erk1/2, p38, and SAPK/JNK. Conversely, the overexpression of RaGAP1 in cells with silenced METTL3 or YTHDF1 partially restored the phosphorylation levels. These findings support the idea that METTL3/YTHDF1 promotes CRC progression through RanGAP1 via the MAPK signaling pathway.

Despite our findings, there are several limitations to our study. Specifically, while we established the MAPK signaling pathway as downstream of RanGAP1, we did not ascertain the specific regulatory mechanism involved. The identification of CRABP2, a protein that co-expresses and interacts with RanGAP1, was achieved through Label-free LC/MS proteomics and co-IP protein MS. It was found that CRABP2 can promote CRC progression via the MAPK signaling pathway. However, subsequent verification revealed that the sequencing result was a false positive, indicating that there is no actual interaction between these two proteins. Previous studies [5, 38, 39] have confirmed the involvement of RanGAP1 in the nuclear pore transport of specific proteins and RNAs, suggesting its potential role in regulating MAPK signaling through CRABP2 transport, which may impact CRC proliferation and metastasis. As a cytoplasmic filament within the nuclear pore complex, RanGAP1 does not necessarily require binding proteins for mediating nuclear access. Therefore, we investigated the disparity between cytoplasmic and nuclear CRABP2 expression in control cells and RanGAP1 knockdown cells using the PARIS kit (Invitrogen, Carlsbad, CA, USA). The findings indicated that knockdown of RanGAP1 had no impact on the nuclear-cytoplasmic distribution of CRABP2 in DLD1 cells. Consequently, we were unable to obtain conclusive experimental evidence confirming RanGAP1’s involvement in regulating CRABP2 entry into or exit from nuclei. Therefore, our laboratory will continue to conduct experiments in order to further investigate the mechanisms underlying the actions of RanGAP1 and CRABP2.

In conclusion, we have identified the oncogenic roles of RanGAP1 in CRC tumorigenesis for the first time. METTL3 enhances RanGAP1 expression through m6A modification by recruiting YTHDF1, thereby regulating CRABP2 and activating the MAPK pathway to promote CRC progression. These findings provide new insights into the diagnosis and treatment of CRC.

### Supplementary information

Supplementary figure 1-5 description

Supplementary table

Supplementary figure 1

Supplementary figure 2

Supplementary figure 3

Supplementary figure 4

Supplementary figure 5

## Data Availability

The datasets used in the current study are available from the corresponding author on reasonable request.
