# Supplementary material for: METTL3-mediated RanGAP1 promotes colorectal cancer progression through the MAPK pathway by recruiting YTHDF1
Source: Cancer Gene Ther. 2024 Jan 24;31(4):562–73. doi: 10.1038/s41417-024-00731-5 (PMC11016466; doi:10.1038/s41417-024-00731-5)
Supplement: Supplementary file 1 — Supplementary figure 1-5 description [file 41417_2024_731_MOESM1_ESM.docx]

**Supplementary figure 1**

**RanGAP1 is highly expressed in CRC.** (A). RanGAP1 mRNA expression levels in various cancer and corresponding normal tissues based on TCGA data by TIMER website. (B, C). RanGAP1 mRNA levels in colorectal cancer and normal intestinal tissue based on GSE49355 and GSE35834 data. (D, E). RanGAP1 mRNA expression levels of patients with primary and metastatic colon cancer based on GSE87211 and GSE21815. (F). RanGAP1 protein levels in colorectal cancer and normal intestinal tissue based on CPTAC website.

**Supplementary figure 2**

**RanGAP1 promotes CRC tumorigenesis in vitro and in vivo.** (A-D). The CCK8 assays and colony formation assays revealed distinct effects on the proliferation of CRC cells upon knockdown or overexpression of RanGAP1. (E, F). Flow cytometry assays showed the change of stably transfecting shRanGAP1 and RanGAP1 on the CRC cell cycle. (G). Xenografts formed by subcutaneous injection of shRanGAP1 DLD1 cells and control cells.

**Supplementary figure 3**

**Screening and oncogenic role of METTL3 in colorectal cancer.** (A). The GO enrichment analyzed RBPs associated with RanGAP1 mRNA. (B-E). The performance of METTL3, METTL5, METTL14 and METTL16 was evaluated in GEPIA, TCGA, KMplot, GSE39582 and GSE161158 datasets. (F). METTL3 expression levels were tested in METTL3 knockdown HCT116 and DLD1 cells by western blotting. (G, H). The change of proliferation ability after silencing METTL3 on CRC cell by CCK8 assays and colony formation assays. (I). The change of migration and invasion abilities after silencing METTL3 on CRC cell by transwell assays. (J). Different density distribution of m6A peaks across mRNA transcripts were identified by MeRIP-seq in METTL3 silenced HCT116 and control group. (K). Prediction results and MeRIP-seq results were combined to obtain the specific site of m6A in RanGAP1. (L). RNA stability of RanGAP1 mRNA in downregulating METTL3 CRC cells and control cells after giving actinomycin D (5 μg/mL).

**Supplementary figure 4**

**Screening and oncogenic role of YTHDF1 in colorectal cancer.** (A, B). The relationship between RanGAP1 and m6A “readers” (IGF2BP1/2/3, YTHDF1/2/3, YTHDC1/2) in CRC. (C, D). RNA stability of RanGAP1 mRNA in YTHDF1 knockdown CRC cells and control cells after giving actinomycin D (5 μg/mL). (E). The changes of YTHDF1 protein levels were tested by western blotting after YTHDF1 knockdown in CRC cells. (F, G). CCK8 assays and colony formation assays showed the effects of silencing YTHDF1 on HCT116 and DLD1 cell proliferation. (H). The migration and invasion abilities were detected in YTHDF1-silenced HCT116 and DLD1 cells with the corresponding control cells by transwell assays.

**Supplementary figure 5**

**The screening of downstream targets of RanGAP1.** (A). Venn diagram showing the proteins regulated by RanGAP1 and the proteins that interact with RanGAP1. (B) Western blot detection of potential downstream molecules in silenced RanGAP1 DLD1 cells. (C) Endogenous binding of RanGAP1 and CRABP2 was investigated by co-IP and western blot. (D) Real-time PCR analysis shows change of CRABP2 mRNA expression levels in RanGAP1 knockdown DLD1 cells.
