# Supplementary material for: METTL3-mediated RanGAP1 promotes colorectal cancer progression through the MAPK pathway by recruiting YTHDF1
Source: Cancer Gene Ther. 2024 Jan 24;31(4):562–73. doi: 10.1038/s41417-024-00731-5 (PMC11016466; doi:10.1038/s41417-024-00731-5)
Supplement: Supplementary file 2 — Supplementary table [file 41417_2024_731_MOESM2_ESM.docx]

| **Supplementary table 1: Relative information of data base used in this study** | | | | | |
| --- | --- | --- | --- | --- | --- |
| **Source** | **Dataset names** | **RNA sequence** | **Platforms** | **Country** | **Last update date** |
| GEO | GSE68468 | Human Genome U133A Array | GPL96 | USA | 2018 |
| GEO | GSE75117 | Microarray data | GPL16699 | Japan | 2020 |
| GEO | GSE21815 | Microarray data | GPL6480 | Japan | 2019 |
| GEO | GSE87211 | Microarray data | GPL13497 | USA | 2018 |
| GEO | GSE161158 | Human Genome U133 Array | GPL570 | USA | 2022 |
| GEO | GSE49355 | Human Genome U133A Array | GPL96 | France | 2018 |
| GEO | GSE35834 | Multispecies miRNA-1 Array | GPL8785 | Italy | 2019 |
| GEO | GSE39582 | Human Genome U133 plus 2.0 Array | GPL570 | France | 2021 |
| TCGA | COAD-US | RNA sequence | Illumina | USA | - |
| TCGA | READ-US | RNA-sequence | Illumina | USA |  |

**Supplementary table 2: Knockdown shRNA sequences used in this study**

| **TRC Number** | **Sequence** |
| --- | --- |
| METTL3-shRNA | Sh1: CCGGGCTGCACTTCAGACGAATTATCTCGAGATAATTCGTCTGAAGTGCAGCTTTTTG  Sh2: CCGGCGTCAGTATCTTGGGCAAGTTCTCGAGAACTTGCCCAAGATACTGACGTTTTTG  Sh3: CCGGGCAAGTATGTTCACTATGAAACTCGAGTTTCATAGTGAACATACTTGCTTTTTG |
| YTHDF1-shRNA | Sh1: CCGGCCCTACCTGTCCAGCTATTACCTCGAGGTAATAGCTGGACAGGTAGGGTTTTTG  Sh2: CCGGACGACATCCACCGCTCCATTACTCGAGTAATGGAGCGGTGGATGTCGTTTTTTG  Sh3: CCGGATCGGTCTAAAGTGCTAATTTCTCGAGAAATTAGCACTTTAGACCGATTTTTTG |
| RanGAP1-shRNA | Sh1: CCGGCCCTACCTGTCCAGCTATTACCTCGAGGTAATAGCTGGACAGGTAGGGTTTTTG  Sh2: CCGGACGACATCCACCGCTCCATTACTCGAGTAATGGAGCGGTGGATGTCGTTTTTTG |
| CRABP2-shRNA | Sh1: CCGGGAAATGGGAGAGTGAGAATAACTCGAGTTATTCTCACTCTCCCATTTCTTTTTG  Sh2: CCGGTCAAACAGGAGGGAGACACTTCTCGAGAAGTGTCTCCCTCCTGTTTGATTTTTG  Sh3: CCGGACAGAGATTAACTTCAAGGTTCTCGAGAACCTTGAAGTTAATCTCTGTTTTTTG |

**Supplementary table 3: Different primer sequences of this study**

| **Primer Name** | **Primer sequences** |
| --- | --- |
| RanGAP1 |  |
| Forward primer: | 5'- GCAGCGTCCAGATGCAGCTC-3' |
| Reverse primer: | 5'- GGGAGAGGCAGGGTCCCCAA-3' |

**Supplementary table 4: Different antibodies used in this study**

| **Antibody name** | **Sources** | **Antibody name** | **Sources** |
| --- | --- | --- | --- |
| METTL3 | proteintech, 15073-1-AP | AKT | Proteintech, 60203-1-Ig |
| m6A | SYSY, Cat. No. 202 003 | P-AKT | Proteintech, 66444-1-Ig |
| YTHDF1 | Proteintech, 17479-1-AP | UBE2I | Proteintech, 10070-1-AP |
| RanGAP1 | Proteintech, 11102-1-AP | LASP1 | Proteintech, 10515-1-AP |
| CRABP2 | Proteintech, 10225-1-AP | BLMH | Proteintech, 14941-1-AP |
| p-ERK | Proteintech, 28733-1-AP | PPIA | Proteintech, 10720-1-AP |
| ERK | Proteintech, 67170-1-Ig | CFL1 | Proteintech, 10960-1-AP |
| p-P38 | CST, 4511T | HMGB1 | Proteintech, 66525-1-Ig |
| P38 | Proteintech, 66234-1-Ig | PFN1 | Proteintech, 11680-1-AP |
| p-SAPK/JNK | CST, 4668S | PGK1 | Proteintech, 17811-1-AP |
| SAPK/JNK | CST, 9252T | GAPDH | Proteintech, 60004-1-Ig |

**Supplementary table 5: Relevant sequences of Luciferase reporter assay in this study**

| **Sequence Name** | **sequences** |
| --- | --- |
| RanGAP1-WT | GCAGCGTCCAGATGCAGCTCGGACATTGGGGACCCTGCCTCTCCC |
| RanGAP1-Mut | GCAGCGTCCAGATGCAGCTCGGCCATTGGGGACCCTGCCTCTCCC |

**Supplementary table 6: The RNA-binding proteins (PBPs) associated with RanGAP1 mRNA**

|  | **RBP** | **geneID** | **geneName** | **geneType** | **clusterNum** | **clipExpNum** | **clipIDnum** |
| --- | --- | --- | --- | --- | --- | --- | --- |
|  | HNRNPC | ENSG00000100401 | RANGAP1 | protein_coding | 44 | 25 | 335 |
|  | TARDBP | ENSG00000100401 | RANGAP1 | protein_coding | 20 | 60 | 276 |
|  | RBMX | ENSG00000100401 | RANGAP1 | protein_coding | 49 | 9 | 217 |
|  | RNPS1 | ENSG00000100401 | RANGAP1 | protein_coding | 20 | 16 | 151 |
|  | ELAVL1 | ENSG00000100401 | RANGAP1 | protein_coding | 33 | 23 | 143 |
|  | ALYREF | ENSG00000100401 | RANGAP1 | protein_coding | 29 | 10 | 138 |
|  | SCAF4 | ENSG00000100401 | RANGAP1 | protein_coding | 33 | 6 | 123 |
|  | SCAF8 | ENSG00000100401 | RANGAP1 | protein_coding | 30 | 6 | 112 |
|  | YTHDF1 | ENSG00000100401 | RANGAP1 | protein_coding | 20 | 15 | 100 |
|  | RBM10 | ENSG00000100401 | RANGAP1 | protein_coding | 26 | 2 | 84 |
|  | DDX3X | ENSG00000100401 | RANGAP1 | protein_coding | 16 | 12 | 78 |
|  | RBFOX2 | ENSG00000100401 | RANGAP1 | protein_coding | 21 | 13 | 70 |
|  | U2AF1 | ENSG00000100401 | RANGAP1 | protein_coding | 35 | 4 | 65 |
|  | PTBP1 | ENSG00000100401 | RANGAP1 | protein_coding | 20 | 12 | 61 |
|  | G3BP1 | ENSG00000100401 | RANGAP1 | protein_coding | 23 | 9 | 60 |
|  | SRSF1 | ENSG00000100401 | RANGAP1 | protein_coding | 20 | 8 | 58 |
|  | RBM15B | ENSG00000100401 | RANGAP1 | protein_coding | 24 | 2 | 54 |
|  | U2AF2 | ENSG00000100401 | RANGAP1 | protein_coding | 18 | 13 | 54 |
|  | IGF2BP2 | ENSG00000100401 | RANGAP1 | protein_coding | 25 | 6 | 53 |
|  | YTHDC1 | ENSG00000100401 | RANGAP1 | protein_coding | 21 | 9 | 52 |
|  | SOX2 | ENSG00000100401 | RANGAP1 | protein_coding | 20 | 2 | 49 |
|  | DDX54 | ENSG00000100401 | RANGAP1 | protein_coding | 14 | 5 | 48 |
|  | SP1 | ENSG00000100401 | RANGAP1 | protein_coding | 19 | 5 | 48 |
|  | CHTOP | ENSG00000100401 | RANGAP1 | protein_coding | 9 | 6 | 45 |
|  | UPF1 | ENSG00000100401 | RANGAP1 | protein_coding | 15 | 13 | 45 |
|  | YTHDF3 | ENSG00000100401 | RANGAP1 | protein_coding | 12 | 6 | 44 |
|  | MTDH | ENSG00000100401 | RANGAP1 | protein_coding | 17 | 1 | 43 |
|  | PRPF8 | ENSG00000100401 | RANGAP1 | protein_coding | 17 | 4 | 41 |
|  | FIP1L1 | ENSG00000100401 | RANGAP1 | protein_coding | 22 | 5 | 40 |
|  | NUDT21 | ENSG00000100401 | RANGAP1 | protein_coding | 23 | 5 | 39 |
|  | CPSF6 | ENSG00000100401 | RANGAP1 | protein_coding | 19 | 4 | 37 |
|  | DHX36 | ENSG00000100401 | RANGAP1 | protein_coding | 6 | 3 | 33 |
|  | EIF4A3 | ENSG00000100401 | RANGAP1 | protein_coding | 16 | 3 | 32 |
|  | YBX1 | ENSG00000100401 | RANGAP1 | protein_coding | 11 | 6 | 31 |
|  | GRSF1 | ENSG00000100401 | RANGAP1 | protein_coding | 6 | 4 | 29 |
|  | METTL1 | ENSG00000100401 | RANGAP1 | protein_coding | 7 | 6 | 29 |
|  | ZCCHC14 | ENSG00000100401 | RANGAP1 | protein_coding | 17 | 2 | 28 |
|  | CTCF | ENSG00000100401 | RANGAP1 | protein_coding | 12 | 3 | 27 |
|  | HNRNPA2B1 | ENSG00000100401 | RANGAP1 | protein_coding | 19 | 3 | 27 |
|  | CPSF7 | ENSG00000100401 | RANGAP1 | protein_coding | 23 | 2 | 26 |
|  | YTHDF2 | ENSG00000100401 | RANGAP1 | protein_coding | 7 | 9 | 26 |
|  | HDLBP | ENSG00000100401 | RANGAP1 | protein_coding | 12 | 2 | 24 |
|  | RBM15 | ENSG00000100401 | RANGAP1 | protein_coding | 19 | 2 | 24 |
|  | HNRNPK | ENSG00000100401 | RANGAP1 | protein_coding | 12 | 7 | 23 |
|  | ILF3 | ENSG00000100401 | RANGAP1 | protein_coding | 11 | 3 | 23 |
|  | RBM4 | ENSG00000100401 | RANGAP1 | protein_coding | 10 | 7 | 22 |
|  | CDK1 | ENSG00000100401 | RANGAP1 | protein_coding | 5 | 2 | 21 |
|  | PCBP2 | ENSG00000100401 | RANGAP1 | protein_coding | 12 | 6 | 21 |
|  | PUS10 | ENSG00000100401 | RANGAP1 | protein_coding | 5 | 6 | 21 |
|  | CSTF2T | ENSG00000100401 | RANGAP1 | protein_coding | 17 | 2 | 20 |
|  | RNF10 | ENSG00000100401 | RANGAP1 | protein_coding | 8 | 1 | 19 |
|  | SRRM4 | ENSG00000100401 | RANGAP1 | protein_coding | 16 | 3 | 19 |
|  | SRSF7 | ENSG00000100401 | RANGAP1 | protein_coding | 15 | 3 | 18 |
|  | TIA1 | ENSG00000100401 | RANGAP1 | protein_coding | 11 | 2 | 18 |
|  | EIF3A | ENSG00000100401 | RANGAP1 | protein_coding | 13 | 2 | 17 |
|  | LIN28B | ENSG00000100401 | RANGAP1 | protein_coding | 7 | 6 | 17 |
|  | NXF1 | ENSG00000100401 | RANGAP1 | protein_coding | 12 | 8 | 15 |
|  | CSTF2 | ENSG00000100401 | RANGAP1 | protein_coding | 8 | 3 | 14 |
|  | CELF2 | ENSG00000100401 | RANGAP1 | protein_coding | 3 | 9 | 13 |
|  | FMR1 | ENSG00000100401 | RANGAP1 | protein_coding | 9 | 4 | 13 |
|  | LARP4B | ENSG00000100401 | RANGAP1 | protein_coding | 4 | 2 | 13 |
|  | MBNL1 | ENSG00000100401 | RANGAP1 | protein_coding | 11 | 1 | 13 |
|  | AQR | ENSG00000100401 | RANGAP1 | protein_coding | 6 | 2 | 12 |
|  | CPSF1 | ENSG00000100401 | RANGAP1 | protein_coding | 10 | 4 | 12 |
|  | ELAVL3 | ENSG00000100401 | RANGAP1 | protein_coding | 4 | 5 | 11 |
|  | TRMT10A | ENSG00000100401 | RANGAP1 | protein_coding | 8 | 2 | 11 |
|  | FAM120A | ENSG00000100401 | RANGAP1 | protein_coding | 7 | 4 | 10 |
|  | MSI1 | ENSG00000100401 | RANGAP1 | protein_coding | 5 | 2 | 10 |
|  | SF3B4 | ENSG00000100401 | RANGAP1 | protein_coding | 7 | 2 | 10 |
|  | WDR4 | ENSG00000100401 | RANGAP1 | protein_coding | 4 | 4 | 10 |
|  | MOV10 | ENSG00000100401 | RANGAP1 | protein_coding | 4 | 3 | 9 |
|  | SRSF9 | ENSG00000100401 | RANGAP1 | protein_coding | 5 | 4 | 9 |
|  | DHX9 | ENSG00000100401 | RANGAP1 | protein_coding | 3 | 4 | 8 |
|  | IGF2BP3 | ENSG00000100401 | RANGAP1 | protein_coding | 5 | 5 | 8 |
|  | ACIN1 | ENSG00000100401 | RANGAP1 | protein_coding | 7 | 1 | 7 |
|  | CAPRIN1 | ENSG00000100401 | RANGAP1 | protein_coding | 6 | 1 | 7 |
|  | DDX21 | ENSG00000100401 | RANGAP1 | protein_coding | 6 | 2 | 7 |
|  | FXR2 | ENSG00000100401 | RANGAP1 | protein_coding | 5 | 3 | 7 |
|  | CENPC | ENSG00000100401 | RANGAP1 | protein_coding | 4 | 2 | 6 |
|  | DAP3 | ENSG00000100401 | RANGAP1 | protein_coding | 4 | 2 | 6 |
|  | EWSR1 | ENSG00000100401 | RANGAP1 | protein_coding | 6 | 3 | 6 |
|  | FTO | ENSG00000100401 | RANGAP1 | protein_coding | 6 | 1 | 6 |
|  | RBFOX1 | ENSG00000100401 | RANGAP1 | protein_coding | 4 | 2 | 6 |
|  | SRSF3 | ENSG00000100401 | RANGAP1 | protein_coding | 2 | 1 | 6 |
|  | TARBP2 | ENSG00000100401 | RANGAP1 | protein_coding | 5 | 1 | 6 |
|  | AKAP1 | ENSG00000100401 | RANGAP1 | protein_coding | 2 | 4 | 5 |
|  | FBL | ENSG00000100401 | RANGAP1 | protein_coding | 4 | 2 | 5 |
|  | FUS | ENSG00000100401 | RANGAP1 | protein_coding | 5 | 4 | 5 |
|  | GRWD1 | ENSG00000100401 | RANGAP1 | protein_coding | 4 | 2 | 5 |
|  | HNRNPM | ENSG00000100401 | RANGAP1 | protein_coding | 4 | 3 | 5 |
|  | RBM7 | ENSG00000100401 | RANGAP1 | protein_coding | 3 | 4 | 5 |
|  | TRA2A | ENSG00000100401 | RANGAP1 | protein_coding | 4 | 2 | 5 |
|  | WDR33 | ENSG00000100401 | RANGAP1 | protein_coding | 5 | 1 | 5 |
|  | EIF4E | ENSG00000100401 | RANGAP1 | protein_coding | 2 | 2 | 4 |
|  | MBNL2 | ENSG00000100401 | RANGAP1 | protein_coding | 2 | 4 | 4 |
|  | RBPMS | ENSG00000100401 | RANGAP1 | protein_coding | 2 | 2 | 4 |
|  | SF3B1 | ENSG00000100401 | RANGAP1 | protein_coding | 3 | 2 | 4 |
|  | SRSF2 | ENSG00000100401 | RANGAP1 | protein_coding | 1 | 4 | 4 |
|  | TRA2B | ENSG00000100401 | RANGAP1 | protein_coding | 2 | 3 | 4 |
|  | CNBP | ENSG00000100401 | RANGAP1 | protein_coding | 3 | 1 | 3 |
|  | CPSF2 | ENSG00000100401 | RANGAP1 | protein_coding | 3 | 1 | 3 |
|  | DGCR8 | ENSG00000100401 | RANGAP1 | protein_coding | 3 | 1 | 3 |
|  | EIF3D | ENSG00000100401 | RANGAP1 | protein_coding | 2 | 2 | 3 |
|  | G3BP2 | ENSG00000100401 | RANGAP1 | protein_coding | 2 | 2 | 3 |
|  | HNRNPH1 | ENSG00000100401 | RANGAP1 | protein_coding | 1 | 3 | 3 |
|  | LARP1 | ENSG00000100401 | RANGAP1 | protein_coding | 1 | 2 | 3 |
|  | MSI2 | ENSG00000100401 | RANGAP1 | protein_coding | 3 | 2 | 3 |
|  | NONO | ENSG00000100401 | RANGAP1 | protein_coding | 2 | 2 | 3 |
|  | NOP58 | ENSG00000100401 | RANGAP1 | protein_coding | 1 | 2 | 3 |
|  | NUDT16L1 | ENSG00000100401 | RANGAP1 | protein_coding | 3 | 3 | 3 |
|  | RNMT | ENSG00000100401 | RANGAP1 | protein_coding | 1 | 2 | 3 |
|  | CPSF3 | ENSG00000100401 | RANGAP1 | protein_coding | 2 | 1 | 2 |
|  | CPSF4 | ENSG00000100401 | RANGAP1 | protein_coding | 2 | 2 | 2 |
|  | DICER1 | ENSG00000100401 | RANGAP1 | protein_coding | 2 | 1 | 2 |
|  | EIF3G | ENSG00000100401 | RANGAP1 | protein_coding | 2 | 2 | 2 |
|  | IGF2BP1 | ENSG00000100401 | RANGAP1 | protein_coding | 2 | 1 | 2 |
|  | MARF1 | ENSG00000100401 | RANGAP1 | protein_coding | 2 | 1 | 2 |
|  | MTA1 | ENSG00000100401 | RANGAP1 | protein_coding | 2 | 1 | 2 |
|  | PABPC4 | ENSG00000100401 | RANGAP1 | protein_coding | 1 | 1 | 2 |
|  | RBM27 | ENSG00000100401 | RANGAP1 | protein_coding | 2 | 2 | 2 |
|  | RTCB | ENSG00000100401 | RANGAP1 | protein_coding | 2 | 1 | 2 |
|  | SF3A3 | ENSG00000100401 | RANGAP1 | protein_coding | 2 | 2 | 2 |
|  | SRSF6 | ENSG00000100401 | RANGAP1 | protein_coding | 2 | 1 | 2 |
|  | SSB | ENSG00000100401 | RANGAP1 | protein_coding | 2 | 2 | 2 |
|  | ALKBH5 | ENSG00000100401 | RANGAP1 | protein_coding | 1 | 1 | 1 |
|  | BCCIP | ENSG00000100401 | RANGAP1 | protein_coding | 1 | 1 | 1 |
|  | BCL11B | ENSG00000100401 | RANGAP1 | protein_coding | 1 | 1 | 1 |
|  | EIF3B | ENSG00000100401 | RANGAP1 | protein_coding | 1 | 1 | 1 |
|  | FUBP1 | ENSG00000100401 | RANGAP1 | protein_coding | 1 | 1 | 1 |
|  | FXR1 | ENSG00000100401 | RANGAP1 | protein_coding | 1 | 1 | 1 |
|  | GTF2F1 | ENSG00000100401 | RANGAP1 | protein_coding | 1 | 1 | 1 |
|  | HNRNPA1 | ENSG00000100401 | RANGAP1 | protein_coding | 1 | 1 | 1 |
|  | KHSRP | ENSG00000100401 | RANGAP1 | protein_coding | 1 | 1 | 1 |
|  | NAT10 | ENSG00000100401 | RANGAP1 | protein_coding | 1 | 1 | 1 |
|  | POLR2A | ENSG00000100401 | RANGAP1 | protein_coding | 1 | 1 | 1 |
|  | PUM1 | ENSG00000100401 | RANGAP1 | protein_coding | 1 | 1 | 1 |
|  | RBM20 | ENSG00000100401 | RANGAP1 | protein_coding | 1 | 1 | 1 |
|  | SLBP | ENSG00000100401 | RANGAP1 | protein_coding | 1 | 1 | 1 |
|  | SMNDC1 | ENSG00000100401 | RANGAP1 | protein_coding | 1 | 1 | 1 |
|  | SND1 | ENSG00000100401 | RANGAP1 | protein_coding | 1 | 1 | 1 |
|  | SNRPA | ENSG00000100401 | RANGAP1 | protein_coding | 1 | 1 | 1 |
|  | TIAL1 | ENSG00000100401 | RANGAP1 | protein_coding | 1 | 1 | 1 |
|  | XRN2 | ENSG00000100401 | RANGAP1 | protein_coding | 1 | 1 | 1 |
|  | YBX3 | ENSG00000100401 | RANGAP1 | protein_coding | 1 | 1 | 1 |
|  | ZC3H7B | ENSG00000100401 | RANGAP1 | protein_coding | 1 | 1 | 1 |
|  | ZNF800 | ENSG00000100401 | RANGAP1 | protein_coding | 1 | 1 | 1 |
