# Supplementary figures and images for: METTL3-mediated RanGAP1 promotes colorectal cancer progression through the MAPK pathway by recruiting YTHDF1
Source: Cancer Gene Ther. 2024 Jan 24;31(4):562–73. doi: 10.1038/s41417-024-00731-5 (PMC11016466; doi:10.1038/s41417-024-00731-5)

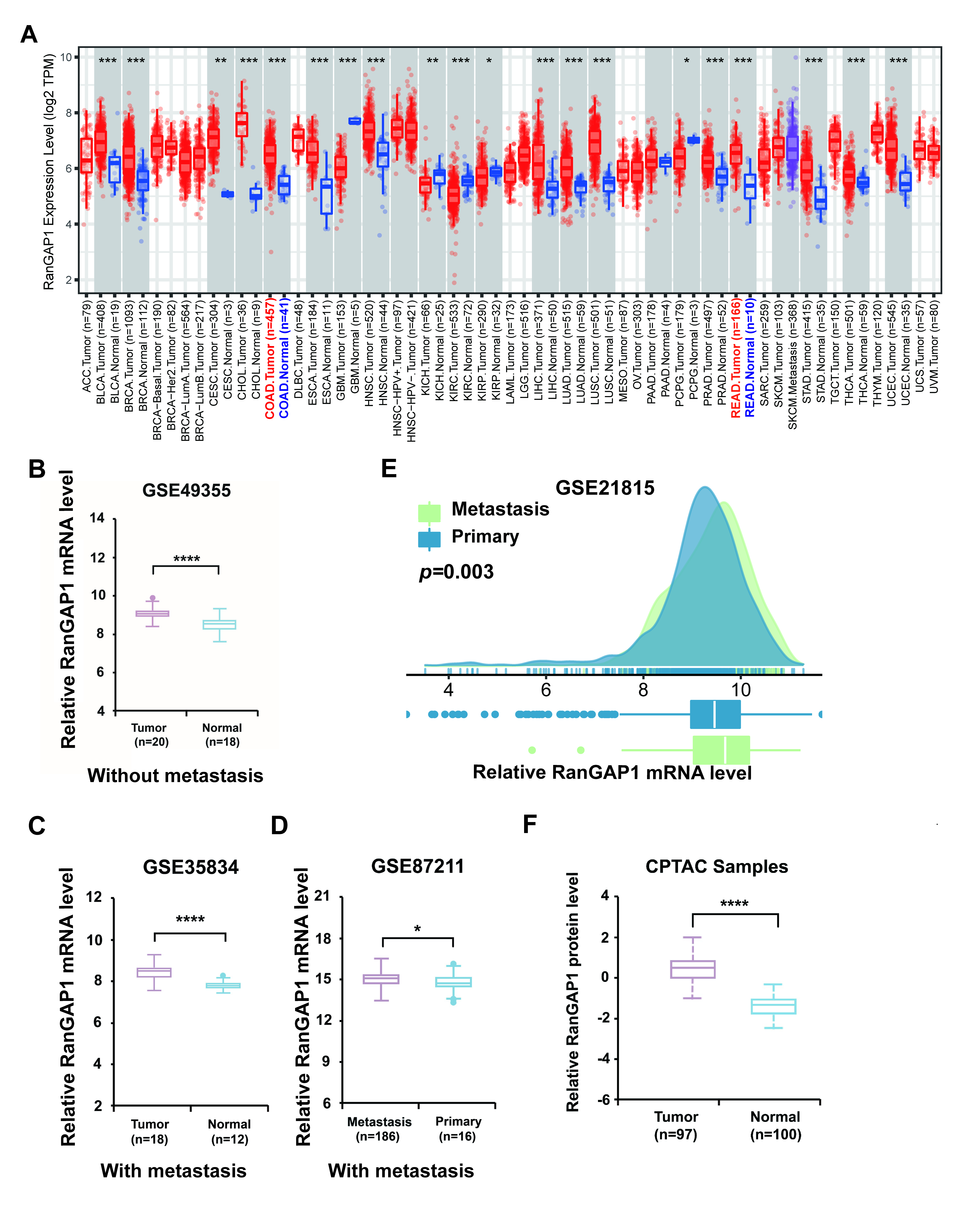

Supplement: Supplementary file 3 — Supplementary figure 1 [file 41417_2024_731_MOESM3_ESM.tif]

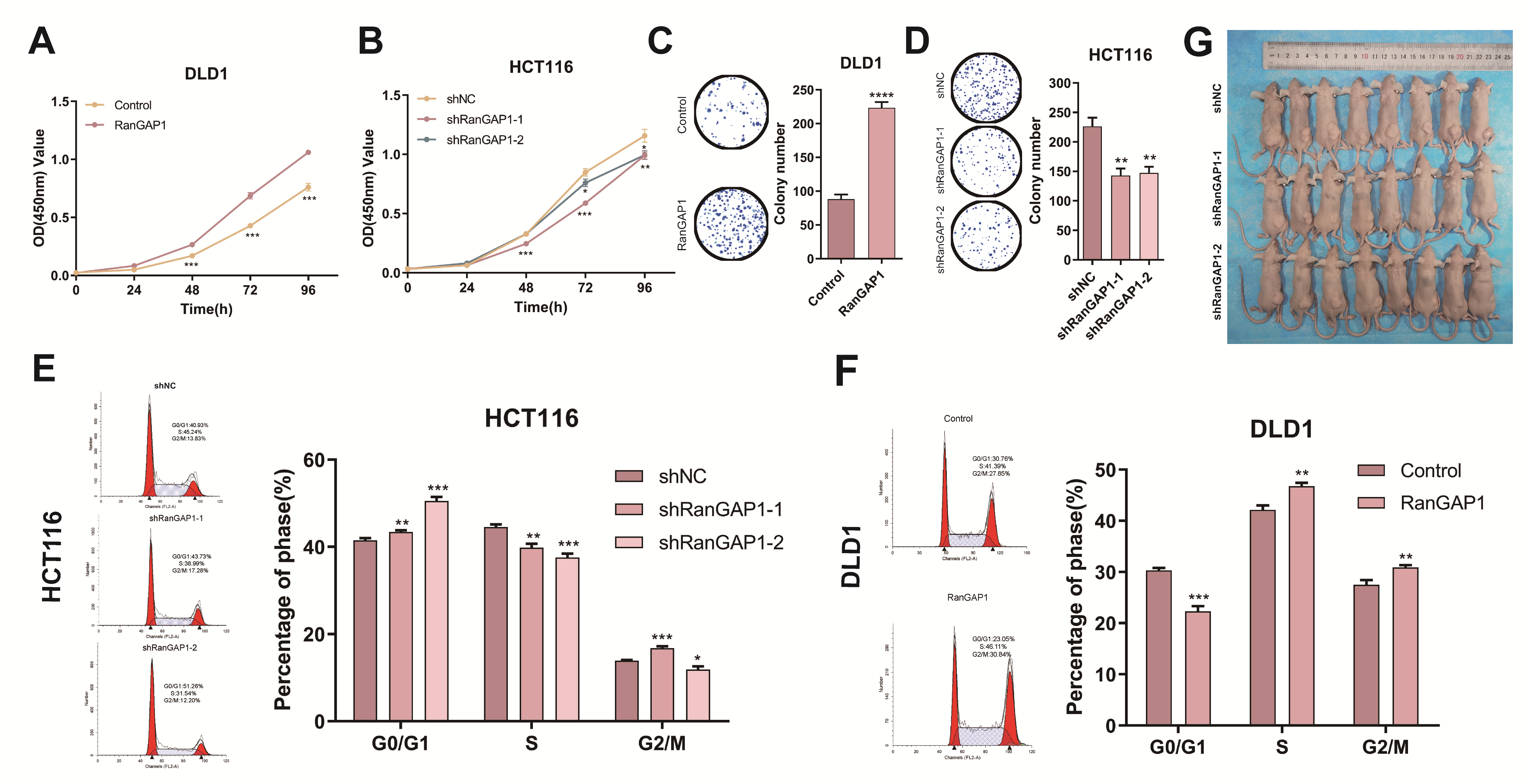

Supplement: Supplementary file 4 — Supplementary figure 2 [file 41417_2024_731_MOESM4_ESM.tif]

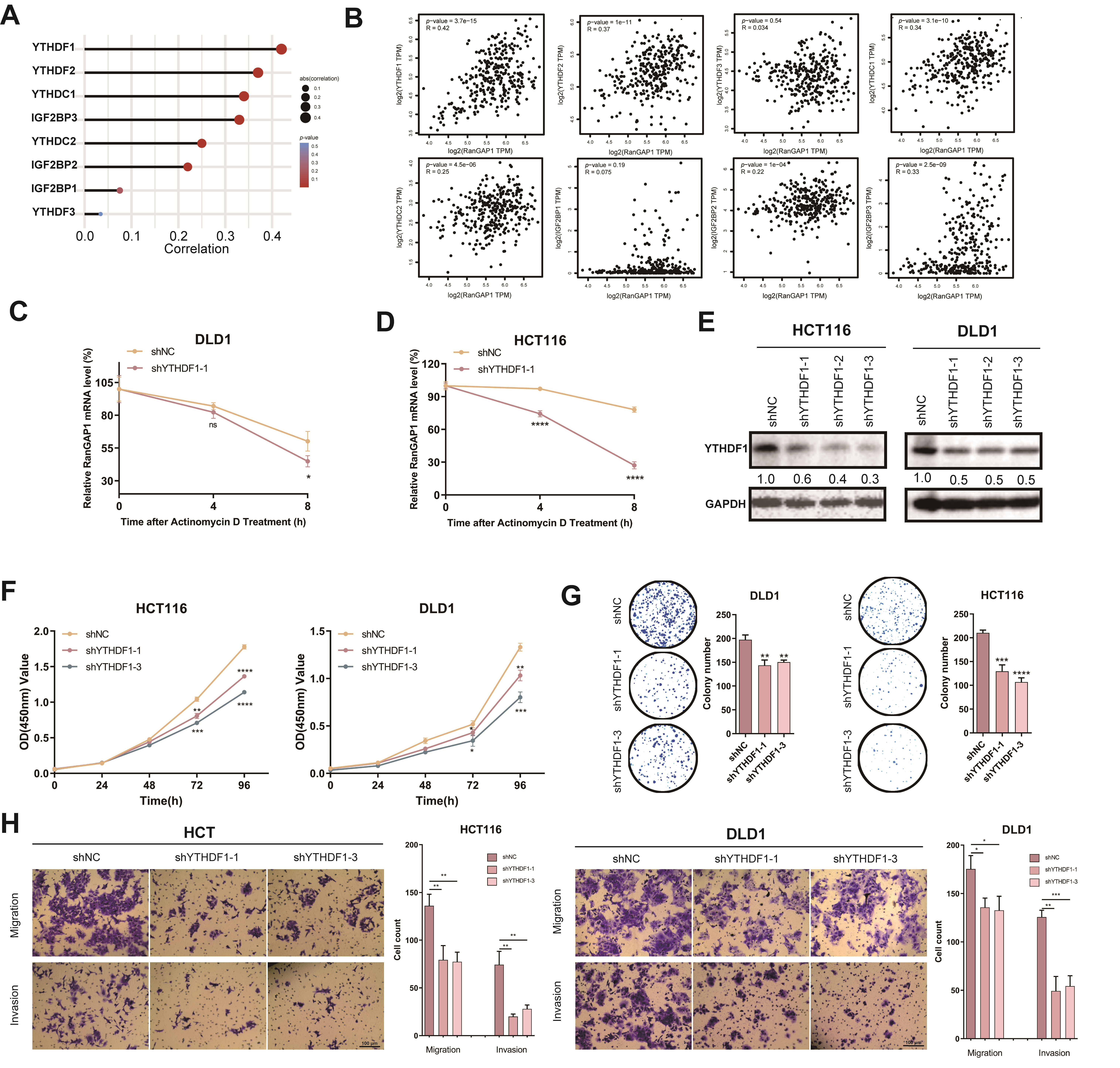

Supplement: Supplementary file 6 — Supplementary figure 4 [file 41417_2024_731_MOESM6_ESM.tif]

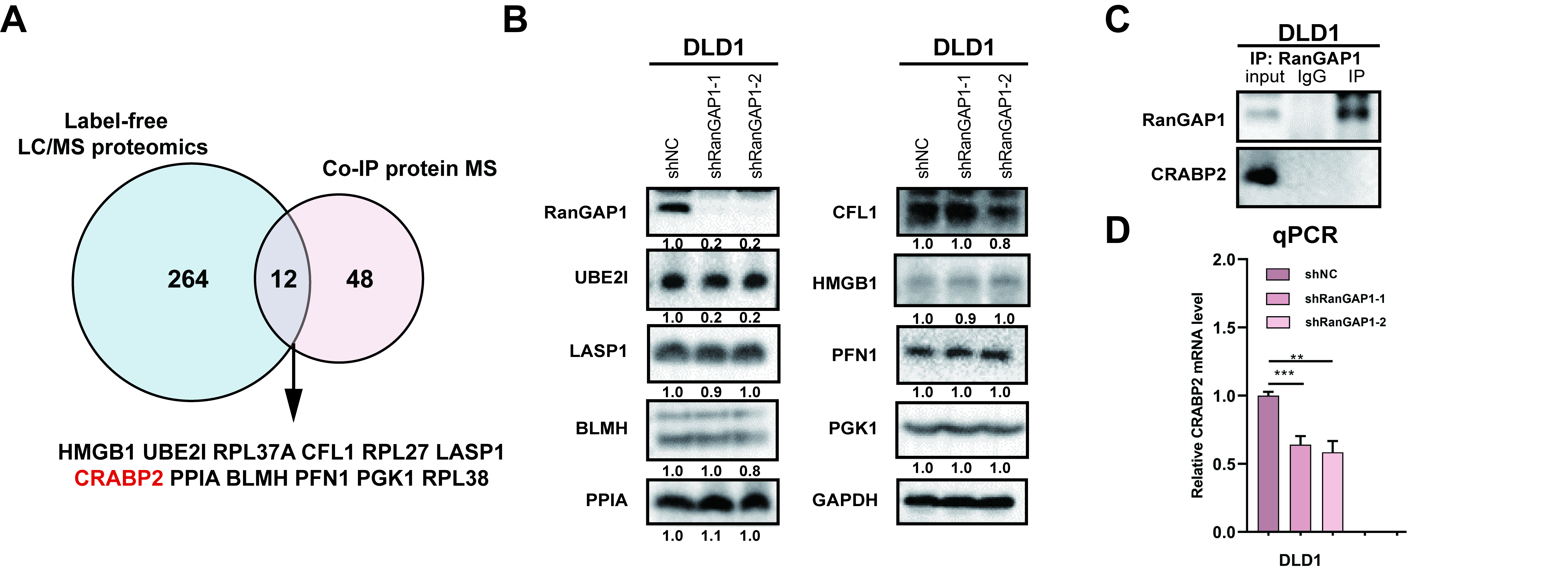

Supplement: Supplementary file 7 — Supplementary figure 5 [file 41417_2024_731_MOESM7_ESM.tif]
